# The Constrained Disorder Principle Overcomes the Challenges of Methods for Assessing Uncertainty in Biological Systems

**DOI:** 10.3390/jpm15010010

**Published:** 2024-12-28

**Authors:** Yaron Ilan

**Affiliations:** Department of Medicine, Hadassah Medical Center, Faculty of Medicine, Hebrew University, Jerusalem 9112102, Israel; ilan@hadassah.org.il; Tel.: +972-2-6776966

**Keywords:** uncertainty, complex systems, noise, digital health, algorithms

## Abstract

Different disciplines are developing various methods for determining and dealing with uncertainties in complex systems. The constrained disorder principle (CDP) accounts for the randomness, variability, and uncertainty that characterize biological systems and are essential for their proper function. Per the CDP, intrinsic unpredictability is mandatory for the dynamicity of biological systems under continuously changing internal and external perturbations. The present paper describes some of the parameters and challenges associated with uncertainty and randomness in biological systems and presents methods for quantifying them. Modeling biological systems necessitates accounting for the randomness, variability, and underlying uncertainty of systems in health and disease. The CDP provides a scheme for dealing with uncertainty in biological systems and sets the basis for using them. This paper presents the CDP-based second-generation artificial intelligence system that incorporates variability to improve the effectiveness of medical interventions. It describes the use of the digital pill that comprises algorithm-based personalized treatment regimens regulated by closed-loop systems based on personalized signatures of variability. The CDP provides a method for using uncertainties in complex systems in an outcome-based manner.

## 1. Introduction

George Edward Box, a well-known statistician who worked in Bayesian inference, quality control, and time-series analysis, is commonly quoted as saying, “All models are wrong, but some are useful”. This statement is highly relevant to models in biological systems. Models that perform well mathematically but lack meaningful clinical outcomes may be irrelevant for everyday clinical work.

The highly complex nature of biological systems challenges applying methods for assessing uncertainty in complex systems. The constrained disorder principle (CDP) defines living organisms based on their inherent variability, which is constrained within dynamic borders [1].

The present paper highlights some challenges in assessing uncertainties in biological systems. It focuses on the intrinsic variability that characterizes these systems and the need to account for their noise when attempting to understand and model them. Tailoring the model to the aim of the analysis is discussed, using new CDP-based second-generation artificial intelligence (AI) systems that incorporate randomness, variability, and uncertainty into their treatment algorithms.

### Definitions

There is much confusion about the definitions used in the literature when investigating system irregularity. Disorder, variability, randomness, noise, and uncertainty are often used interchangeably, and scientists from different disciplines sometimes use these terms with different meanings.

Some suggested definitions are based on commonly used sources [2,3,4,5,6].

- **Noise** implies having too many variables in any area that prohibit clear linkages between them.
- **Disorder** refers to a lack of order.
- Variability implies a natural variation and a change over time or being subjected to variation or changes.
- **Randomness** is the unpredictability concerning measurement. It is incomplete knowledge that can be quantified. A state is random without a definite plan, purpose, or pattern.
- **Functional randomness** is defined in cases where noise is constructive, leading a system toward robustness.

Both randomness and noise present a limit to predictability.

vi. **Uncertainty** refers to an incomplete or lack of knowledge and degree of accuracy with which a quantity is measured.
vii. **Irregularity or variability** implies not being or acting according to laws, rules, or established customs and not conforming to the usual inflection pattern.
viii. **Chaos** refers to a state of utter confusion. Due to its sensitivity to small changes in conditions, it appears random because of its unpredictable behavior.
ix. **Entropy** refers to the regularity of the data and measures the rate at which information is gained. An orderly and regular system has a reduced entropy.
x. **Heterogeneity** is the quality or state of being diverse in character or content.

## 2. The Importance of Accounting for Uncertainty in Biology

### 2.1. Why Is Accounting for Uncertainty and Randomness Critical in Biological Systems?

Uncertainty characterizes most complex systems. Even after the exclusion of classical fluctuations, models of complex systems still comprise uncertainty [7]. Collecting data on any complex system’s noise and error models is an essential part of the information required for a better understanding of the system [7]. Being an inherent part of biological systems makes randomness, variability, and uncertainty critical to recognize and account for when modeling these systems. Understanding uncertainty is essential for risk management decisions about these systems. Modeling and predicting biological processes mandate accounting for the uncertainties and randomness that characterize them. It improves models’ outputs to enable better significant results in the clinical setting [8,9,10].

### 2.2. The Two Types of Uncertainty

Models that account for uncertainties are schematically divided into two types.

- **Data noise, randomness in the phenomenon, missing data, error, and measurement noise cause aleatoric uncertainty**. Sometimes, it refers to statistical and systematic uncertainty that cannot be reduced by improving the model or expanding the dataset. Image uncertainty, for instance, is not reduced by a more extensive dataset but can be enhanced by it. Improved measurements and reduced error or noise mitigate this uncertainty. A high aleatoric uncertainty can be attributed to a wrong prediction, while a low aleatoric uncertainty is attributed to a correct prediction [11,12,13].
- **Epistemic uncertainty** is due to a lack of data. It refers to a lack of understanding or knowledge of an input data point and estimation errors. In the training dataset, every observable data point and the approximation error are included, so no model can perfectly approximate the unknown actual function. An example is uncertainty due to the image quantity, which is reduced by having more data, increasing the model complexity, and increasing the sample used to train the model [14,15,16].

These two types of uncertainties are relevant in biological processes, where the lack of data and the noise of the systems contribute to uncertainties. As built-in randomness and uncertainties characterize biological systems, reducing the two uncertainties is possible but not eliminated.

### 2.3. Some Challenges Faced When Modeling Biological Systems

Developing models that account for biological systems’ inherent uncertainty and randomness is mandatory for applying them to clinical settings [17]. The modeling of biological systems involves many unknown parameters and has a complex structure, which requires a substantial amount of evaluation time relative to the analysis needs [18].

Since multiple parameters and platforms can control biological systems, unraveling the regulatory relationships is challenging and requires determining how biological information is communicated [19,20]. The actual proper signal in biological systems is partly uncertain. The statistical model is a way to express uncertainty about the outcome of an experiment. However, the model itself may be wrong. Bioinformatics, genome sequencing, proteomic, metabolomic, and other techniques significantly increased biological data. One of the challenges in these systems is predicting how interactions between molecular components impact cells’ functions. Models of nonlinear dynamics of complex interactions are designed to analyze these data [18]. A kinetic model describes each biological reaction based on its corresponding biological process, and a differential equation describes the properties of the entire system [21,22,23].

Refining the model mandates a set of systematic uncertainties and an understanding that uncertainty and randomness are part of the system [24,25]. Noise analysis is required to improve biological systems’ modeling, and correcting errors in these models depends on how coherent the noise process is [26]. Models of biological processes such as immunoreceptor signaling require parameterization and uncertainty quantification to make reliable predictions and account for the noise using the relevant error models [7,27]. Whenever a model is analyzed, searching for parameters over a high-dimensional parameter space that incorporates the appropriate sources of uncertainty is necessary [18]. In the absence of acceptable points, the model is inconsistent with the observed data and based on incorrect biological principles. Incorporating observation error and model discrepancy terms simplifies the global parameter search and smooths the likelihood surface, making it more robust. It reduces the likelihood of following a misleading global minimum [18].

Most systems biology models aim to explain a natural system’s behavior. A crucial aspect of assessing the adequacy of such models is to compare them with experimental data, which involves uncertainties. It is necessary to look into acceptable matches while avoiding false conclusions based on a limited number of runs to determine a sufficient match between a model run and the observed data. Such findings can change if an alternative run matches the data but provides different biological implications [18,28,29]. Using models for predicting biological experiment results requires finding all acceptable matches and the corresponding range of predictions studied. If a narrow range of forecasts from the acceptable runs for a particular future experiment is found, then it could be a test of the model as it could rule it out.

Conversely, an extensive range implies that the experiment is informative for the model’s rate parameters. Analyses fail due to a lack of an acceptable match between the model and reality or the pursuit of a scientifically false match parameter setting [18]. Similarly, developing models that predict epidemic spread with awareness and heterogeneous transmission rates in networks is challenging [30].

The relevant questions for biological systems are the following: Can the model match the observed data, and if so, what is the entire set of input parameter choices that give acceptable matches between the model output and observed data? Uncertainties in inputs and outputs must be accounted for when comparing a model to observed data and statistically analyzing the association between the model and reality. The time required to evaluate the model numerically in these systems may be extended, making it not feasible [18].

Models with numerous uncertain parameters pose a significant challenge as these parameters cannot be directly measured. Additionally, parameters measured in experimental conditions may differ from those in the cellular environment. Therefore, comparing the model’s outputs with experimental observations, such as measured concentrations and trends, is crucial to determine which input or rate parameter values match the model and reality [18]. This process involves considering several sources of uncertainty, including the observation error, biological variability, and tolerance for refining the model’s accuracy, also known as the model discrepancy. A global parameter search for all input parameter settings is necessary to achieve an acceptable match, as a single solution for the rate parameter values may lead to misleading implications and predictions for future experiments. However, global parameter searches are challenging, given the complex structure of the model that imposes constraints on the parameters [31,32].

Table 1 presents several variables contributing to the complexity of modeling biological systems and their inherent uncertainty and randomness [18,27,33,34,35,36,37,38,39,40,41,42]. These variables make modeling systems in biology a fundamental challenge.

## 3. Obtaining a Parameterized Model Consistent with Data in Biology

Different methods and software tools have been developed to address the problem of uncertainty.

Examples are the immunoreceptors on T and B lymphocytes that are part of the information signaling transfer network, which activate numerous processes inside and between cells [43,44]. Models can predict the function of these systems, providing means for quantifying immune cell signaling dynamics [43,44]. However, these models have challenges as one or more unknown rate constants characterize each network interaction. A model for known protein–protein interactions is associated with numerous unknown parameters. These models must quantify uncertainty to provide a more accurate prediction. As the number of different chemical species increases, these simulations become computationally demanding [27].

Models are created in a standardized format to ensure compatibility with commonly used tools such as Data2Dynamics, COPASI, AMICI, PyBioNetFit, and PESTO [45,46,47,48,49,50,51,52]. Most modeling systems assume a compartmental model is constructed based on known molecular signaling. Immunoreceptor signaling models use BioNetGen language (BNGL), which employs rule-based modeling to describe biomolecular dynamics. For a broader range of software support for rule-based modeling, the Systems Biology Markup Language (SBML) is used [53]. It is beneficial to use standardized formats because of the availability of databases such as the Bio Models Database archives [53,54,55]. A model that describes the concentrations of populations of chemical species over time comprises nonlinear ordinary differential equations (ODEs), which generate deterministic time courses for each chemical species. Alternatively, it could be a stochastic model simulated using Gillespie’s direct method or a network-free method, where the state is followed based on a set of molecules present in the system without counting for all possible chemical species and reactions [54].

Measurement models typically assume that experimental data are available and that they relate to the quantities represented in the model. The data usually consist of quantitative time courses or dose–response curves. A function measures how well the model fits with the experimental data, and the parameterization problem involves minimizing the chosen objective function. Some methods are available to minimize this objective function, such as optimization algorithms that provide point estimates for parameter values. Parameter estimation requires the incorporation of both quantitative and qualitative data [27]. Parameter values are estimated using gradient-based or gradient-free methods. Uncertainty quantification uses profile likelihood, bootstrapping, or Bayesian inference [27].

i. Optimization based on gradients involves calculating the gradient of the objective function concerning the parameters. These gradients can be first-order, which only uses the first derivative of the aim function concerning the parameters, or second-order, which uses both the first and second derivatives. First-order methods include gradient descent and stochastic gradient descent. Second-order methods, like the Levenberg–Marquardt algorithm, are not hindered by saddle points and can use the curvature information in the second derivatives. However, computing gradients for systems biology is not a straightforward process [56]

The finite-difference approximation is a simple approach that estimates the gradient by slightly altering each variable. On the other hand, the forward sensitivity method provides an accurate calculation of the gradient. This method involves adding extra variables to the original ordinary differential equation (ODE) system to represent the derivative of each species concentration concerning each parameter. The forward sensitivity method requires solving an ODE system of sensitivity equations for each parameter to compute the gradient. Each sensitivity equation system has the same size as the original ODE model. However, calculating the gradient can be expensive when the problem has multiple parameters and ODEs, which could limit its feasibility [57,58]. Adjoint sensitivity analysis simplifies the problem by combining the original integration with the backward integration of a newly derived system. However, this method cannot handle non-trivial initial conditions [58,59,60]. Automatic differentiation involves propagating the derivatives of each operation’s products via the graph using the chain rules [61,62].

The limitation of all gradient-based optimization algorithms is that they can become stuck at the objective function’s local minima or saddle points. To overcome this problem, multiple replicates of optimization starting from different initial points, termed multi-start optimization, may be used [27].

ii. Metaheuristic optimization algorithms operate by repeated objective function evaluations without using gradient information to find a global optimum. The algorithms keep a group of appropriate parameters and create fresh parameter sets for testing [32]. There are two types of algorithms: asynchronous and synchronous. Asynchronous algorithms help with load balancing by running multiple simulations simultaneously. These algorithms select new trial parameter sets with limited new data. On the other hand, synchronous algorithms require all simulations of one iteration to be completed before moving on to the next iteration. Both asynchronous and synchronous algorithms use multiple cores to reduce the time required for fitting [27,63,64]. Metaheuristic algorithms can be helpful for problems where gradient-based methods fail, such as estimating parameters in stochastic models for TCR signal initiation analyzed by network-free simulation [65,66].

The algorithms assume a quantitative objective function. However, some methods enable the practical analysis of non-numerical, qualitative data [67,68,69]. Qualitative data can be represented as constraints that limit the outputs of a model. These constraints are inequalities integrated into the model’s objective function as penalty functions. When a constraint is met, the penalty function has a value of zero, but when a constraint is violated, the penalty function takes on a value proportional to the degree of the violation [27]. PyBioNetFit has introduced the Biological Property Specification Language (BPSL) to define inequality constraints on the model’s outputs. BPSL defines the qualitative properties of time courses and dose–response curves observed experimentally [27].

## 4. Modeling the Uncertainty in Systems

The previously described methods assist in obtaining a parameterized model consistent with data. In biological systems, the model must account for uncertainty in parameter estimates, which propagate to uncertainty in the predictions [27]. This type of analysis is helpful in high-dimensional parameter spaces with limited experimental data or multiple hidden variables that impact the system and cannot be identified. Uncertainty quantification assesses confidence in parameter values and model predictions [70]. Improved modeling methods estimate and compute uncertainties with diverse datasets to reduce standard error [71,72,73,74,75]. The emergent behavior of generic dynamical systems under random environmental perturbations was described.

When the set is characterized by uncertainty and randomness, machine learning (ML) provides probabilities but is limited in its ability to reach complete accuracy.

The neural network output uncertainty should be validated by interpreting the associated probabilities and comparing the test set with the frequency of correct answers [10,76,77,78,79]. The uncertainty in the prediction set may lead to a mistake, or the algorithm can understand that two different animals are close to each other [80].

Noise in these systems is computed into a distribution. There are models where denoise and decoder are used to remove noise and where denoiser and encoder are used [81,82,83]. In models of molecule structures, where many atoms are in noisy distribution, they can be denoised into a stable molecule. Denoising molecule diffusion assists in getting into a final structure by generating positions and getting the atoms to interact with the atoms around them and be put in for the proper connection [84]. In other models, noise can be added to the system and processed [85,86,87,88]. It implies summing up distributions of different molecules. The emergent behavior of generic dynamical systems under random environmental perturbations was described [89]. Table 2 describes several methods for modeling of uncertainty.

- **Profile likelihood** is a method used to determine the identifiability of a model’s parameters. It is performed by scanning a parameter of interest over a series of fixed values. At each fixed value, the optimization of the objective function is repeated, allowing the values of all other free parameters to vary. The objective function value is then plotted against the fixed parameter value. Similarly, prediction uncertainty can be calculated using this method. It is important to note that the objective function must be related to a statistical likelihood function that determines the probability of generating experimental data for the chosen model and parameterization. This method helps quantify the identifiability of individual parameters and model predictions but does not provide information about parameter relationships [90,91,92].
- **Bootstrapping** is a statistical method that helps to deal with parameter uncertainty by resampling data. However, this method should be carefully used when coping with non-identifiable parameters. The available dataset was first resampled several times to perform bootstrapping. Then, the optimization algorithm is applied to each resampled dataset, and the best fit is saved as a bootstrapped parameter set. This process is repeated numerous times to generate the desired number of bootstrapped parameter sets, which help us determine confidence intervals for each parameter. Resampling the data is believed to be equivalent to repeating the experiment. However, if the optimization algorithm is biased, it can lead to biased bootstrap estimates of confidence intervals. Therefore, it is essential to ensure the optimization algorithm is unbiased to obtain accurate results [93,94,95].
- **Single-network deterministic methods** use a single neural network that provides a deterministic prediction. An additional network addresses uncertainty in classification problems, where multiple answers may exist for a single input point. For instance, if different physicians provide different diagnoses for the same image, the model computes the corresponding frequency of positive and negative answers, and the networks are trained on these frequencies. Another approach is to use an evidential neural network, an extension of density neural networks that uses a probability distribution on the probability distribution parameters [96,97,98,99,100].
- **Ensemble methods** involve training multiple neural networks to provide output variability. It includes random initialization of network parameters, shuffling the database, and incorporating random noise in gradient descent. It requires learning and storing several models [101,102,103].
- **Test-time augmentation methods** are used when training a model that expands the training set with modified copies of samples from the training dataset. The artificially expanded training dataset results in a more skillful model, assisting in extracting and learning features. It can introduce noise or distortion in the input to obtain variability in the prediction, Gaussian noise, or other noises. However, it involves selecting the noise level and the type of distortions [104,105,106]. Using nuisance parameters or adding variability to the known and unknown model may increase the uncertainties for the parameters of interest [107,108].
- **Bayesian statistics** typically address parameter uncertainty rather than noise. It involves obtaining a sample according to an unknown distribution. This method considers model parameters as random variables with unknown probability distributions. The multivariate probability distribution of the parameters is calculated based on experimental data to quantify uncertainty. The Bayesian approach combines the likelihood with the prior distribution to form a posterior distribution proportional to their product. When describing posterior variance, it is essential to distinguish it from noise; the posterior variance represents uncertainty, not noise.

Examining the parameter’s marginal distribution and assessing the correlations between parameters can determine a credible interval from the resulting distribution for each parameter. Additionally, prediction uncertainty can be quantified by examining simulation outputs for sampled parameter sets [70]. This method provides a way to obtain probabilistic answers by generating a posterior distribution for all uncertain variables. It requires a prior specification of joint probability distributions for all variables and assumes only one valid variable exists. It also assumes that the statistical model used to define the variable is accurate. The posterior distribution gives the most probable values for the valid input variable [18].

Bayesian statistics overcome the challenge of global parameter searches and the uncertainty analysis of complex models [18,109,110]. These models construct emulators, which are statistical constructs that mimic the scientific model, providing predictions with uncertainty at unevaluated input parameter settings [18,28,110,111,112,113,114]. The emulator is fast to evaluate, provides an understanding of the structure, and assists in performing a global parameter search [28,115,116,117]. An emulator is a tool that attempts to replicate the behavior of a complex model. Its purpose is to predict the model’s output at a specific input that has not been tested yet and provide a measure of uncertainty for that prediction in the form of posterior distributions. By doing this, emulators can give us an idea of what to expect and how confident we can be in our predictions for untested inputs. Emulators are tested for their accuracy in predicting the model’s behavior over a new batch of runs. It helps us identify regions of the input space that are unlikely to occur and determine which input parameters are acceptable [18].

Observational or experimental errors are the most identifiable sources of uncertainty. It is essential to differentiate between a model and a biological system. The model discrepancy allows for the integration of additional information about its shortcomings to enhance the model’s prediction [18,118,119].

**The Monte Carlo dropout technique** involves deactivating noise on neurons or weights. It can be performed for weights where each component follows a Bernoulli or Gaussian distribution. The Metropolis–Hastings (MH) Markov Chain Monte Carlo (MCMC) algorithm improves the sampling of multimodal distributions. If gradient information is available for the problem, Hamiltonian Monte Carlo (HMC) can be used instead. Emulators can deal with the many model evaluations required for MCMC algorithms, but this comes at the expense of increased uncertainty [27,120,121,122,123,124,125,126,127,128].

A Bayesian approach is relevant for cases where detailed calculations are necessary, in well-tested, accurate biological models where data with a reliable observation error structure and probabilistic results are vital. This method can be challenging when dealing with familiar distributional forms, such as the multivariate normal distribution, because the likelihood function, a crucial component of Bayesian calculations, is constructed using all the relevant outputs. However, when dealing with outputs that behave erratically, it becomes difficult to emulate them, which can cause issues with emulator diagnostics. Even small changes to the Bayesian specification related to the likelihood can result in significant changes to the posterior. These sensitivities can be challenging to detect and analyze, but they can raise concerns about the conclusions drawn from the results [18,129,130,131,132].

## 5. The Bayesian History Matching for Determining Uncertainties

When comparing a systems biology model with the observed data, we must ensure that the input parameters generate acceptable matches. To check this, we use a method called “history matching”, which assesses whether the model fits the data and identifies areas in the input parameter space that lead to acceptable matches. This technique uses “improbability measures” to determine areas of the input space that can be eliminated from further investigation. It requires only a limited specification of uncertain quantities, such as means, variances, and covariances. By using history matching, we can forecast future experiment results and make informed decisions accordingly [18,28].

Bayesian emulators are models that replicate the behavior of a complex model while being computationally efficient. They are integrated into an iterative history-matching process that can explore high-dimensional spaces while accounting for various sources of uncertainty. This Bayesian history-matching approach has been successful across multiple scientific fields, including cosmology, epidemiology, oil reservoir modeling, climate modeling, environmental science, and traffic modeling. By investigating the input rate parameter space, this method provides valuable insights into the underlying structure of the model, the constraints imposed on it by observed data, and the resulting biological implications. It also evaluates the results obtained from a secondary history match of an alternative model. If the input space is deemed improbable, the model is inconsistent with the observed data regarding the specified uncertainties. It could mean that the biological principles underlying the model are incorrect, or there is an underestimation of observation errors, model discrepancy, or emulator uncertainty. When a more significant model discrepancy is required, it suggests that the model is inaccurate. Bayesian emulation and history matching can determine the uncertainty of quantitative trends in regulatory relationships, hormonal signaling systems, and gene functions; however, trends of biological data are not sufficiently quantitative [18,28,29,133,134].

A recent study provides an example for modeling complex systems biology using Bayesian emulation methodology for uncertainty analysis [18]. The described method incorporates sources of uncertainty, such as observational errors and model discrepancy, to establish links to reality. It uses an emulator to quickly explore the behavior of a model, which can be applied to systems with long evaluation times. Using improbability measures that perform a global exploration of the input parameter space, a history match can be performed to identify the region containing all acceptable matches to the observed data. This method models the difference between the model outputs and the observed data, including sources of uncertainty such as observational errors and model discrepancy [18].

The activities of auxin, ethylene, and cytokinin in plant biology depend on the cell context in which they are present and exhibit interactions that can either work together or against each other. A network of hormonal interactions in a single Arabidopsis cell was analyzed using kinetic modeling to understand how it regulates auxin in the root by controlling the contribution of auxin influx, biosynthesis, and efflux and integrating auxin, ethylene, and cytokinin signaling. The model was constructed based on known molecular interactions and experimental evidence, with some parameters based on experimental data and others adjusted to fit the data. The study used Bayesian emulation and history matching to identify a set of all acceptable matches between model outputs and experimental data, considering the primary sources of uncertainty. The research helps link the model to reality and compares the strengths and weaknesses of history matching to more standard approaches, representing probabilistic specifications or expectations and variance statements [18,133].

## 6. Models Involving Causality in Biological Systems

Algorithms for cancer prediction are examples of challenges faced when developing causality models [135]. Prediction models comprising Bayesian networks predict causal relationships between features for probabilistic reasons under uncertainty for clinical decisions [135]. Unlike regression, Bayesian networks define interactions between variables that underlie tumor recurrence. These networks make inferences on any parameters, serving as tools for diagnosis and prognosis [136,137,138].

Tumor recurrence is a significant concern for cancer survivors. Patient variability partly explained the difficulty in predicting tumor recurrence or other disease-related prognoses. A treatment regimen can be associated with being recurrence-free for one patient while not providing a similar effect in another patient [135]. In a recent study, the performance of algorithm-based and expert-based systems was above the chance level. The algorithmic-based structure was more performant and less interpretable than the expert-based structure. Bayesian networks are helpful for clinical applications as they can probabilistically reason under uncertainty while intuitively interpreting the results. The algorithm used in this study captures the process leading to cancer recurrence by connecting variables based on when they are clinically available. While the algorithm-based structure had higher discriminating power than the expert structure, it also contained clinically meaningless relationships due to the challenge of determining causality from the data.

Additionally, using the algorithmic structure carries the risk of overfitting and unnecessarily increasing model complexity. On the other hand, the expert structure was more credible despite its relatively lower predictive performance. In this study, the expert structure did not perform better than the algorithmic structures. Still, it was more clinically suitable as it was calibrated for all time points and had fewer biases. However, using the expert’s structure could introduce an inherent bias based on the expert’s prior knowledge and subjective experience. The data suggest that the expert structure is better calibrated for clinical applications than the algorithmic structure [135,139,140].

Algorithmic structures use parent-to-child connections that are not necessarily aligned with the clinical process and decision making. Systems require a certain level of clinical understanding from experts who can better understand the correlation between variables and causality. There is controversy around which variables need to be included in results from patients’ heterogeneity in different studies. It is not always necessary to base the inclusion of variables in a prediction model strictly on algorithms, even if it enhances performance.

## 7. The Constrained Disorder Principle Provides a Scheme for Dealing with Uncertainty and Randomness in Biological Systems

### 7.1. Using the CDP to Deal with System Uncertainty

The constrained disorder principle (CDP) defines biological systems by their intrinsic randomness [1]. According to the CDP, this randomness must be kept within dynamic, personalized boundaries. This principle ensures that biological systems remain flexible and adaptable to constantly changing internal and external environments. Intrinsic uncertainty, randomness, and variability are all essential for the proper functioning of living organisms [1,141,142,143,144,145].

The CDP is schematically described by the B = F formula, where B stands for borders and F for systems functionality and efficiency. According to this formula, biological systems’ boundaries are fundamental for proper system function by adjusting the degree of variability. This property ensures adaptation to dynamic internal and external perturbations [146].

Per the CDP, biological systems possess a mixture of order and disorder and require a certain degree of disorder and uncertainty for function under perturbations [147,148]. Randomness is intrinsic to biological systems, from the genome to organ function, leading to unpredictability and heterogeneity [141,142,143,144]. Uncertainty and variability are essential for the proper function of systems and are not background noise [1].

According to the CDP, an organism is not a straightforward process but a continuous progression through states far from equilibrium. The coexistence of opposite properties, which results in a combination of stability and instability, creates a blend of order and disorder within ordered systems. It means that such systems exhibit both structural strength and non-conservative behavior. Regarding systems development, it is proposed that the dynamic instability of biological systems comprising different forms of randomness, variability, and uncertainty can reflect part of the organization along with evolution [141,142,143,144,147,148,149,150,151,152,153,154,155,156,157].

### 7.2. The Objective of Modeling Biological Processes Determines the Type of Calculation to Be Used

There can be different aims for modeling complex biological systems. In cases where modeling aims to understand these systems better, the methods described above provide essential insights into processes. However, these models cannot overcome the inherent uncertainties, randomness, and variabilities underlying these systems’ proper function. Based on the CDP, constrained uncertainty, randomness, and variability characterize systems. It implies that the hidden variables are profound in number and weight, making it impossible to reach sufficient accuracy. Therefore, achieving complete accuracy in modeling to match reality is unreachable.

Clinical decision making is a process where approximation may not always be sufficient. A personalized treatment decision-making process is required in routine clinical settings, which is challenging to predict from a big data-based algorithm. The n = 1 concept was proposed as an option for overcoming this problem in newly developed second-generation algorithms [158,159,160]. While learning from big data sets, these systems consider each patient the sole endpoint for a personalized algorithm [158].

Inputs used for modeling always have a degree of stochasticity. We continuously sample from variable data in modeling, and modeling is always about sampling random particles. The final result is a distribution or multiple distributions. The probability of interactions, which provide essential insight into processes and assist in decision making, is insufficient for complete accuracy. In cases where an approximation is insufficient clinically, modeling of uncertainties can help but cannot serve as a sole final decision-maker [161].

### 7.3. CDP Defines System Malfunctions, Providing a Means for Improving System Functionality

According to the CDP, variability is essential for proper functioning. The dynamic boundaries determine the level of variability and adjust it in response to internal and external changes. It suggests that regulating the level of system variability, whether by increasing or decreasing it, can help address system malfunctions. As a result, disease states are characterized by inappropriate levels of variability [21,24,25]. It was shown for neurological diseases [36,37,38], metabolic disease [32,33,34,40], inflammatory diseases [39,41,42,43], malignant disorders [44], and rare diseases [45].

The CDP may provide a method for enhancing system functionality and efficiency by managing biological variability. It may involve dynamically adjusting the degree of variability to help the system adapt to its fluctuating environments [144,145].

### 7.4. Using Uncertainty and Randomness: CDP-Based Second-Generation AI Systems for Improving Biological Systems Efficiency

Modeling uncertainty and randomness can only lead to a certain degree of accuracy, which can be sufficient under some clinical conditions and insufficient under others. Recently developed algorithms have attempted to use the quantification of uncertainty and randomness [158].

Introducing noise into systems using test-time augmentation methods that introduce distortion in the input to obtain variability in the prediction can improve modeling accuracy. However, they are not meant to keep the noise in the studied system in reality. Bayesian optimization frameworks, such as the evolutionary Monte Carlo sampling, utilize a pattern of defects in an engineered micro-lattice to increase its energy density before it eventually fails. A recent study has shown that the optimized version of the micro-lattice has a normalized strain energy density that is more significant than its defect-free counterpart, typically the focus of research. This platform may be helpful for architected materials and optimization problems showing how defects can enhance the mechanical performance of architected materials [32,162].

Introducing uncertainty, randomness, and variability into biological systems is being developed to improve diagnostics and response to chronic therapies [58,146,153]. About a third of patients suffering from chronic disease develop tolerance to their drugs and partially or entirely lose the effectiveness of these products. The digital pill comprises any drug regulated by a CDP-based second-generation AI system that implements variability in the dosing and administration times to overcome the loss of response [159].

The algorithm is based on quantifying variability signatures and incorporating them into the treatment regimens in a personalized closed-loop system that tailors the treatment plan to the patient [158,159,163]. Implementing the system can improve the outcome of chronic diseases, including hypertension, cancer, chronic pain, heart failure, inflammatory disorders, epilepsy, and rare diseases [23,26,27,29,154].

The system continuously quantifies variability signatures, such as heart rate, blood pressure, gait, genome, and proteome, implementing them into personalized treatment regimens in a personalized way [160,164,165,166,167,168,169].

In patients with heart failure who developed resistance to diuretics, using a CDP-based platform that introduces variability into treatment regimens has led to significant clinical and laboratory improvement and reduced hospitalizations due to heart failure [170]. In patients with cancer, the CDP-based platform overcame drug tolerance, leading to substantial clinical improvement and laboratory improvement [171]. A similar beneficial effect was shown in subjects suffering from genetic disorders, multiple sclerosis, Gaucher disease, and chronic pain [146,172].

Variability-based CDP platforms can be a foundation for creating more accurate digital twins and enhancing diagnostic and therapeutic tools across various medical fields [1,146,158,159,160,163,173,174,175]. They are based on the concept that variability is necessary for proper functioning, and the lack of sufficient variability or out-of-range variability is associated with system malfunction.

### 7.5. The Three Stages of Incorporating Signatures of Variability into CDP-Based Platforms

The second-generation AI system based on CDP is being developed in three stages. The first stage introduces variability into the output within a predefined range in an open-loop system. An example of this is the digital pill, which utilizes variability in the timing and dosages of drug administration to overcome drug resistance. In the second stage, the closed-loop system adjusts the degree of variability to achieve the desired outcome [158,159].

In the third step, we quantify the signatures of variability, which are then used to enhance the accuracy of the algorithm output. These signatures can include physiological variabilities such as heart rate, blood pressure, or variations in blood test analytes.

This three-step method assesses how the variability-based algorithm impacts a system’s desired outcome. It also offers a way to distinguish between physiological and pathological noise, which, according to the CDP, may be indicated by lower or higher system variability [158,159].

## 8. Summary

Biological processes are characterized by uncertainty, randomness, and variability. When modeling biological systems, it is essential to account for these factors. The type of calculation used should be tailored to the goal of the analysis. Current systems are not sufficient for decision making as they require complete accuracy. Second-generation AI systems based on the CDP provide a framework that uses variability signatures to improve output accuracy. These systems are expected to lead to more precise diagnostic and therapeutic regimens. Future studies are needed to customize the platform for additional indications and assess its potential effectiveness in improving system functionalities.

## Figures and Tables

**Table 1:** Variables contributing to modeling biological systems’ complexity, inherent uncertainty, and randomness [18,27,33,34,35,36,37,38,39,40,41,42].

Variable:	Potential Role in Systems Complexity
The inherent uncertainty, randomness, and variability.	Lack of deterministic processes.
The uncertainty involves multiple hidden variables with high weights.	Prohibit simple analysis of processes.
Complex systems have background noise or fluctuations.	Difficulty in distinguishing them from the inherent disorder.
Background noise is part of the system’s function.	Modeling should account for it, not eliminate it.
The number of variables contributing to uncertainty and randomness can be significant.	Many are unknown, and there is a lack of tools to account for them.
Models may account for one or more compartments.	Each may be considered homogeneous and lose clinical relevance if the uncertainty is not considered.
The input problem consists of the variability of the data within some constraints.	Sampling a limited amount of data, errors, and hidden variables reduces accuracy.
Rare events.	May exert a profound effect on systems’ outcomes.
Improving the accuracy of measurements improves conclusions.	It is impossible to eliminate uncertainty and randomness even by increasingthe number of repetitions and using highly accurate measuring tools.
Models are insufficient to care for systems with an exact target label.	Multiple regression controls for multiple standard errors do not resolve it.
Conventional optimization techniques may become stuck in local minima.	It prevents the exploration of small regions of scientific interest within input space.
When modeling complex biological systems, parameterization tools dealwith high-dimensional search spaces.	It minimizes the number of expensive model simulations.
Parameterization and uncertainty.	Their quantification is mandatory for the analysis of quantitative models.

**Table 2:** Several methods for modeling uncertainty of systems.

Modeling Method:	Description of the Method:
Profile likelihood	Scanning a parameter of interest over a series of fixed values is a method for determining the identifiability of a model’s parameters.
Bootstrapping	A statistical method that helps to measure uncertainty by resampling data. The available dataset is resampled several times. Then, the optimization algorithm is applied to each resampled dataset, and the best fit is saved as a bootstrapped parameter set. The process is repeated numerous times to generate the desired number of bootstrapped parameter sets.
Single-network deterministic method	It uses a single neural network that provides a deterministic prediction.
Ensemble methods	Training multiple neural networks to provide output variability by randomly initializing network parameters, shuffling the database, and incorporating random noise in gradient descent.
Test-time augmentation methods	They are used when training a model that expands the training set with modified copies of samples from the training dataset. The expanded training dataset results in a more skillful model, assisting in extracting and learning features.
Bayesian statistics	Obtaining a sample according to an unknown distribution while considering model parameters as random variables with unknown probability distributions. The multivariate probability distribution of the parameters is calculated based on experimental data to quantify the uncertainty.

## Data Availability

Not applicable.

## Abbreviations

CDP	constrained disorder principle;
AI	artificial intelligence;
ML	machine learning;
ODE	ordinary differential equation.
